# Secondary Cardiorenal Syndrome in a Cohort of Septic Patients Treated in a Medical Intensive Care Unit: A Single-Center Experience

**DOI:** 10.7759/cureus.93639

**Published:** 2025-10-01

**Authors:** Bojan C Mitrovic, Ratko S Tomasevic, Svetozar R Mijuskovic, Zoran M Gluvic

**Affiliations:** 1 Gastroenterology and Hepatology, University Clinical-Hospital Centre Zemun, Belgrade, SRB; 2 Internal Medicine, Faculty of Medicine, University of Belgrade, Belgrade, SRB; 3 School of Medicine, University of Belgrade, Belgrade, SRB; 4 Endocrinology, Diabetes and Metabolism, University Clinical-Hospital Centre Zemun, Belgrade, SRB

**Keywords:** cardio-renal syndrome, critical care, infection, internal medicine, sepsis

## Abstract

Context

Sepsis is a life-threatening condition caused by an abnormal host response to infection, resulting in multiple organ failure and high mortality rates. Secondary or type five cardiorenal syndrome (CRS) emerges as a consequence of systemic infection, resulting in both cardiac and renal dysfunction. The synergistic impact of inflammation and hemodynamic instability results in an adverse cycle of vital organs damage during which renal impairment further aggravates cardiac dysfunction and vice versa.

Objectives

The objective of this study is to assess the influence of specified demographic, clinical, and laboratory parameters on the hospitalization outcome of patients who fulfilled CRS-5/Sepsis-3/KDIGO (Kidney Disease: Improving Global Outcomes) criteria treated in the intensive care unit (ICU) from March 2022 to September 2024 (30 months).

Methods

The observational study comprised 41 patients treated at Zemun University Clinical Hospital's medical ICU (mICU). The inclusion criteria were a positive blood culture, elevated procalcitonin (Pct) levels, and clinical and laboratory evidence of heart and renal failure in the presence of sepsis. The cohort of patients was divided into two groups (survivors and deaths) based on hospitalization outcomes. Epidemiological (gender, age, duration of hospital stay), clinical (systolic blood pressure (SBP) and diastolic blood pressure (DBP), ejection fraction (EF), Acute Physiology and Chronic Health Evaluation II (APACHE II) score, the presence of disseminated intravascular coagulation (DIC) and the use of renal replacement therapy (RRT)), and laboratory (white blood cells (WBC) and platelet (Plt) counts, the levels of C-reactive protein (CRP), D-dimer, Pct, urea, creatinine, B-type natriuretic peptide (BNP), troponin (Tn)) parameters were examined. The obtained data were analyzed using the statistical package SPSS for Windows 22.0.

Results

The study cohort had an average age of 70 years (43-84 years), with 22 (54%) female patients. Out of 41 patients, 14 (34%) survived and 27 (66%) died. The values of SBP and APACHE II score, as well as the duration of hospital stay, statistically differed between the two groups (p=0.009, p=0.000, and p=0.000, respectively) and had a significant impact on hospitalization outcome (p=0.007, p=0.000, and p=0.000, respectively).

Conclusion

According to the findings, patients with sepsis and type five CRS had a significant mortality rate (66%). Low SBP, a higher APACHE II score, and a shorter duration of hospital stay are significant predictors of unfavorable outcomes. Additional research and the development of clinical guidelines are required to improve the prognosis of such patients.

## Introduction

Sepsis is a life-threatening condition that is caused by a dysregulated host response to infection, leading to organ dysfunction and high mortality rates, especially in critically ill patients treated in medical intensive care unit (mICU) [1,2]. Among the numerous adverse effects of sepsis, the simultaneous development of cardiovascular and kidney impairment, known as secondary or type five CRS, is an important clinical issue [3-5]. Type five CRS is associated with sepsis and septic shock, resulting in both cardiac and renal dysfunction [4,5].

The incidence of sepsis in Europe has been steadily increasing, with recent data showing that between 2010 and 2020 it ranged from 400 to 800 new cases per 100,000 people per year, with a greater frequency among hospitalized patients [6,7]. Sepsis is especially common in mICUs, where 10%-15% of hospitalized patients develop septic shock [8,9].

Type five CRS has a complicated natural history, with several interdependent processes contributing to organ injury triggered by systemic infection. Excessive cytokine synthesis and release, including tumor necrosis factor-alpha (TNF-α), interleukin-1 (IL-1), and IL-6, result in systemic inflammation and endothelial dysfunction [9-11]. Plasma IL-6 levels predominantly correlate with kidney injury, while TNF-α levels correlate with both kidney and cardiovascular injury [12,13]. Furthermore, neurohormonal disharmony, referring to the maladaptive overactivation of the renin-angiotensin-aldosterone system, sympathetic nervous system, and vasopressin release, leading to vasoconstriction, sodium and water retention, and fibrosis, further compromises kidney and cardiovascular function [14-17]. Additionally, oxidative stress and mitochondrial injury contribute to cellular apoptosis and energy depletion, further exacerbating organ dysfunction [18,19]. As a result, sepsis causes severe hemodynamic changes such as hypotension, reduced cardiac output, and increased vascular permeability [20]. Sepsis-induced microcirculation changes are common, making the cardiovascular and kidney failure more profound [21]. The subsequent hypoperfusion further deteriorates kidney function, whereas myocardial depressants and mitochondrial dysfunction contribute to life-threatening septic cardiomyopathy [22,23]. The synergistic effects of inflammation and endothelial dysfunction promote an adverse cycle of vital organ damage, a bidirectional deterioration of cardiac and renal function in which impairment of one organ accelerates the dysfunction of the other through hemodynamic, inflammatory, and neurohormonal pathways [24,25].

Type five CRS in septic patients is an important diagnostic and management challenge. Biomarkers such as cytokines, troponin (Tn), B-type natriuretic peptide (BNP), and kidney function indicators (creatinine and blood urea nitrogen) are commonly increased, indicating the extension of multiorgan involvement [26-28]. The timely identification of type five CRS in sepsis is critical for improving patient outcomes. The initial assessment should include conventional hematology and biochemistry tests, urinalysis, the measurement of BNP, Tn, and cystatin C levels, as well as imaging studies examining kidney and heart function [28-30]. Current management options include hemodynamic preservation, infection control, and supportive care enabled by vasopressor medication, fluid resuscitation, and renal replacement therapy (RRT) to reduce organ damage [31-33]. Emerging diagnostic and therapeutic options, such as research on novel biomarkers for early risk stratification, anti-inflammatory medications, and extracorporeal blood purification techniques, present intriguing opportunities for improving outcomes in septic patients with type five CRS [28,31,33,34].

Despite the growing body of literature on sepsis-related cardiorenal syndrome (CRS‑5), there is a lack of prospective, single-center data from middle-income countries applying standardized KDIGO and Sepsis‑3 criteria. Furthermore, limited evidence exists on easily obtainable clinical predictors of outcome in this population. This paucity of standardized, prospective data from middle-income countries has been highlighted in recent global and regional studies [35,36]. To address these gaps, we conducted a prospective observational study in a tertiary care ICU over a 30‑month period, focusing on patients who fulfilled CRS‑5/Sepsis‑3/KDIGO (Kidney Disease: Improving Global Outcomes) criteria.

Objectives

The objective of this study is to assess the influence of specified demographic, clinical, and laboratory parameters on the hospitalization outcome of patients who fulfilled CRS-5/Sepsis-3/KDIGO criteria treated in the ICU during a 30-month period [37].

## Materials and methods

Study population

The observational cohort study was conducted at the mICU of the Department of Internal Medicine, University Clinical-Hospital Centre Zemun, Faculty of Medicine, University of Belgrade, Belgrade, Serbia, from March 2022 to September 2024 (30 months). The Ethics Committee of University Clinical-Hospital Centre Zemun-Belgrade approved the study (approval number 65/1 dated July 15, 2025), and it was conducted following the code of ethics of the World Medical Association (Declaration of Helsinki), published in the *British Medical Journal *(July 18, 1964). The study enrolled 41 patients who matched the following basic inclusion criteria: at least one positive blood culture (BC) or quick polymerase chain reaction (PCR) test with antibiogram, high Pct levels as a septic marker, and clinical and laboratory evidence of heart and kidney failure. All patients and family members were informed of the research procedure and signed the medical record. Exclusion criteria were cancer patients (with or without histological proof), aged <18 years and >85 years. The entire study is structured according to Strengthening the Reporting of OBservational studies in Epidemiology (STROBE) principles.

Epidemiological (gender, age, the duration of hospital stay), clinical (systolic blood pressure (SBP) and diastolic blood pressure (DBP), ejection fraction (EF), Acute Physiology and Chronic Health Evaluation II (APACHE II) score, the presence of disseminated intravascular coagulation (DIC) and the use of RRT), and laboratory (white blood cells (WBC) and platelet (Plt) counts, the levels of C-reactive protein (CRP), D-dimer, Pct, urea, creatinine, BNP, Tn) parameters were examined. The entire set of mentioned parameters and procedures is achievable in middle-income countries. Hospitalization outcome is the endpoint of patients’ follow-up reflecting the final discharge status (survive or pass away).

Study protocol

The study protocol included the clinical examination of patients after fulfilling the basic inclusion criteria (blood cultivation provided by the BD Bactec FX TOP device (Becton, Dickinson and Company, Franklin Lakes, NJ, USA), and the antibiogram by using the Vitek 2 Compact device (bioMerieux, Marcy-l'Étoile, France) and signing of the medical records by the patients or family member. Systolic and diastolic blood pressures were measured according to standard protocol by a Riva-Rocci Sphygmomanometer CK-101 (Spirit Medical Co. Ltd., New Taipei City, Taiwan) with a precision of 1 mmHg. Hematological parameters (WBC and Plt counts) were determined on the automatic analyzer Beckman Coulter DxH-900 (Beckman Coulter, Brea, CA, USA). The levels of CRP, urea, and creatinine were measured by the biochemical analyzer Beckman Coulter DxC Au 700 and Au 480 (Beckman Coulter). The measurement of BNP and Pct, as well as high-sensitive Tn, was carried out by Hitachi Cobas Pure (Roche, Basel, Switzerland) and Beckman Coulter Access2 (Beckman Coulter), respectively. D-dimer value was determined by using ACL Top 350 (Werfen, Barcelona, Spain). The entire set of laboratory parameters was determined by commercial tests according to the manufacturer’s instructions.

The APACHE II score is among the most frequently used mICU scoring systems that assess the severity of disease presentation and the risk of death. It is applied within 24 hours of admission of a patient to the mICU and ranges from 0 to 71. The higher scores correspond to more severe disease presentation and a higher risk of death (https://www.mdcalc.com/calc/1868/apache-ii-score) [38]. Ejection fraction (%) was measured by heart ultrasound performed by a single, experienced ultrasonography specialist using HealthCare Vivid E9 XDClear (General Electric, Boston, MA, USA).

CytoSorb cartridges (Cytosorbents Corporation, Princeton, NJ, USA) were placed in a pre-filter position on the AK 200 dialysis machines (Gambro, Lund, Sweden). They were coupled as intermittent hemodialysis (IHD) through transient jugular vascular access. The set of polysulfone (Helixone®, Fresenius Medical Care, Bad Homburg, Germany) membrane filters and unfractionated heparin was used. Blood flow was set to 200-250 ml/min and dialysate flow was between 300 and 500 ml/min. CytoSorb hemoadsorption was selected as an adjunctive therapy in patients with severe sepsis or septic shock who fulfilled CRS‑5 criteria and exhibited markedly elevated circulating inflammatory mediators, with the aim of reducing cytokine burden and mitigating multi‑organ injury. This therapy is particularly relevant to type five CRS because it targets systemic inflammation, a key driver of both cardiac and renal dysfunction in this syndrome [39,40]. CytoSorb IHD was discontinued in patients whose vasopressor drug requirement decreased <20% of the initial dose or if the patient’s clinical condition was not improving [41].

Statistical methods

The methods of descriptive and analytical statistics were used. Methods of descriptive statistics included the measures of central tendency and variability. Analytical statistics included tests for assessing the significance of correlations and differences. Spearman’s rank correlation test was used to determine the correlation’s significance. To determine the significance of the difference, the T-test for independent samples was used in the case of parametric data, and the χ^2^ test in the case of categorical, i.e., the Mann-Whitney U test in cases of interval data that do not follow the normal distribution. Multivariable logistic regression would be used to define predictors of unfavorable outcomes. The level of statistical significance was 0.05. The obtained data were analyzed using the statistical package SPSS for Windows 22.0 (IBM Corp, Armonk, NY).

## Results

**Figure 1:**
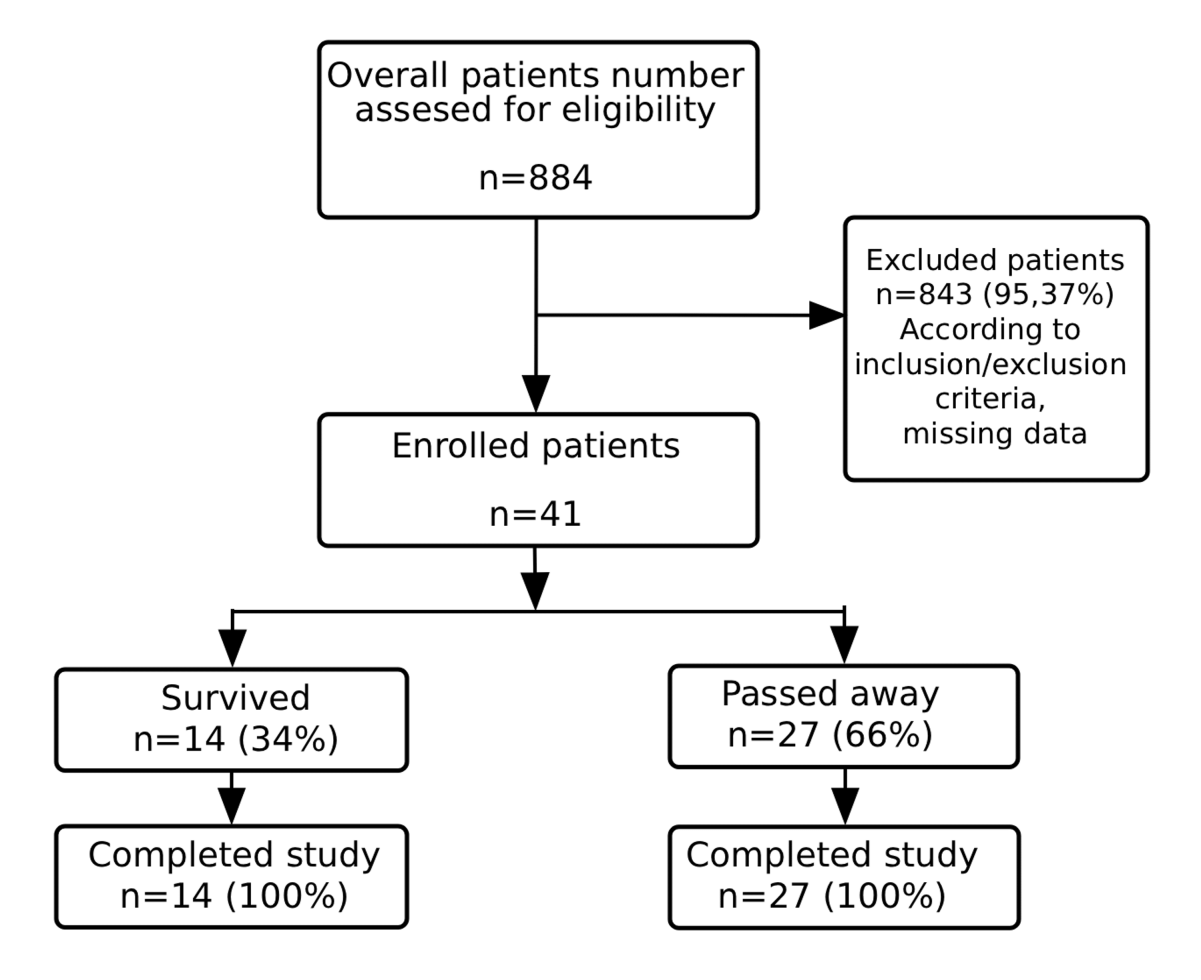
Study consort-type flow diagram.

The average age of the study population is 70 years (43-84 years), with 22 women (54%). The examined population showed no statistically significant difference with regard to age (U=185.000, p=0.912) or gender (χ^2^=0.114, p=0.735). Out of 41 patients, 14 (34%) had a favorable hospitalization outcome, whereas 27 (66%) died. Tables 1, 2 show descriptive statistics for clinical and laboratory parameters in the study cohort. Figure 1 presents the study protocol in a consort-type flow diagram.

**Table 1:** Clinical parameters in the study population SBP: Systolic blood pressure; EF: ejection fraction; DIC: disseminated intravascular coagulation; HD: hemodialysis; APACHE II: Acute Physiology and Chronic Health Evaluation II. T-test (t), chi-square (χ), and Mann-Whitney U tests.

Parameters	Survived (n=14)	Passed away (n=27)	All (n=41)	p
SBP (mmHg) (X±SD (Min-Max))	97±13 (60-105)	81±10 (65-105)	84±12 (60-105)	t=2.745, p=0.009
EF (%) (Med (Min-Max))	47 (28-60)	45 (15-50)	45 (15-60)	U=50.500, p=0.221
DIC (Yes (%))	1 (7)	8 (30)	9 (22)	χ^2^=2.721, p=0.099
HD (Yes (%))	7 (50)	7 (26)	14 (34)	χ^2^=2.376, p=0.123
APACHE II score (Med (Min-Max))	48 (26-78)	78 (50-91)	69 (26-91)	U=28.000, p=0.000
Duration of hospital stay (days) (X±SD (Min-Max))	30±9 (20-50)	12±10 (1-32)	18±13 (1-50)	t=5.353, p=0.000

**Table 2:** Laboratory parameters in the study population CRP: C-reactive protein; Pct: procalcitonin; WBC: white blood cells count; Plt: platelets count; Cr: creatinine; Tn: Troponin; BNP: B-type natriuretic peptide T-test (t), Mann Whitney (U) test.

Parameters	Survived (n=14)	Passed away (n=27)	All (n=41)	p	Reference Range
CRP (mg/L) (X±SD (Min-Max))	287.4±120.5 (41.5-466.5)	249.4±119.0 (54.8-559.3)	262.4±119.4 (41.5-559.3)	t=0.964 p=0.341	< 5.0
D dimer (ng/mL) (Med (Min-Max))	3048 (614-71283)	3229 (416-48730)	3133 (416-71283)	U=178.500 p=0.773	0-230
Pct (ng/mL) (Med (Min-Max))	13.9 (1.0-100.0)	17.0 (1.0-100.0)	15.2 (1.0-100.0)	U=180.000 P=0.804	< 0.5
WBC (x10^9^/L) (Med (Min-Max))	15.1 (3.5-57.2)	14.7 (1.3-47.5)	15.1 (1.3-57.2)	U=188.000 p=0.978	3.4-9.7
Plt (x10^9^/L) (X±SD (Min-Max))	186±88 (30-312)	139±133 (3-560)	155±121 (3-560)	t=1.192 p=0.241	150-450
Urea (mmol/L) (Med (Min-Max))	18.5 (7.8-41.0)	20.8 (6.7-92.2)	18.6 (6.7-92.2)	U=181.500 p=0.837	2.8-7.2
Cr (μmol/L) (X±SD (Min-Max))	401±187 (138-663)	332±167 (134-644)	356±175 (134-663)	t=1.206 p=0.235	59-104
Tn (ng/L) (Med (Min-Max))	263 (93-9600)	279 (23-18892)	279 (23-18892)	U=125.000 p=0.801	<18.3
BNP (pg/mL) (X±SD (Min-Max))	20441±12572 (5697-35000)	17638±16269 (212-40000)	18573±14610 (212-40000)	t=0.300 p=0.770	<125.00

A significant correlation has been identified between hospitalization outcome and SBP (ρ=-0.415, p=0.007), APACHE II score (ρ=+0.701, p=0.000), and the duration of hospital stay (ρ=-0.622, p=0.000). Furthermore, there is no statistical significance between hospitalization outcome and demographic (age and gender) or other clinical parameters (EF value, presence of DIC, receiving hemodialysis (HD)). Table 3 shows the Spearman correlation coefficients between the hospitalization outcome and the observed laboratory parameters (CRP, D-dimer, Pct, WBC, Plt, urea, creatinine, Tn, and BNP). Table 4 shows the predictivity of the clinical parameters for the hospitalization outcome. A list of the microbes cultured in blood is presented in Table 5.

**Table 3:** The correlation of the hospitalization outcome and laboratory parameters CRP: C-reactive protein; Pct: Procalcitonin; WBC: white blood cells; Plt: platelets; Cr: creatinine; Tn: Troponin; BNP: B-type natriuretic peptide. Spearman’s rank (ρ) correlation test.

Parameters	CRP	D dimer	Pct	WBC	Plt	Urea	Cr	Tn	BNP
Hospitalization outcome (ρ)	-0.174	-0.046	-0.039	-0.004	-0.313	+0.033	-0.202	-0.044	-0.103
p	0.277	0.777	0.808	0.978	0.046	0.849	0.205	0.805	0.749

**Table 4:** Logistic regression model of clinical predictors for the hospitalization outcome SBP: Systolic blood pressure; OR: odds ratio; CI: confidence interval; SE: standard error; APACHE II: Acute Physiology and Chronic Health Evaluation II.

Parameters	B	SE	Wald	OR (95% CI)	p
SBP	0.047	0.61	0.601	1.049 (80-88)	0.438
APACHE II score	0.197	0.08	5.619	1.214 (60-72)	0.018
Duration of hospital stay	-0.255	0.128	3.939	0.775 (14-22)	0.047

**Table 5:** The blood-cultured microbes in ICU patients

Microbe	n	%
Escherichia coli	1	2.4
Acinetobacter	5	12.2
Streptococcus pneumoniae	4	9.8
Staphylococcus epidermidis	7	17.1
Coagulase-negative *Staphylococcus*	7	17.1
*Klebsiella* spp.	4	9.8
Staphylococcus hominis	4	9.8
Staphylococcus aureus	5	12.2
*Pseudomonas* spp.	1	2.4
Stenotrophomonas maltophilia	1	2.4
Staphylococcus haemolyticus	1	2.4
Streptococcus agalactiae	1	2.4
Total	41	100

## Discussion

Sepsis is a systemic infection followed by the host's systemic inflammatory response syndrome (SIRS) [1]. The rising incidence of septic conditions results from increased life expectancy, a higher number of invasive diagnostic and therapeutic procedures, increased pathogen virulence and invasiveness, and their developing resistance to antimicrobial treatment [2,3,6,12]. A number of septic conditions are complicated by the failure of vital organs, primarily the heart, vascular bed, and kidneys, complicating patients' already serious clinical presentation and final outcome. These confounding factors constitute essential diagnostic components of type five CRS and predict poor outcomes in patients [4,5,10].

This study examined the influence of demographic, clinical, and laboratory parameters on the hospitalization outcome of patients with type five CRS treated in a university hospital's mICU over a two-year period. Our study's findings suggest a high death rate (66%) among these patients, which is consistent with prior research indicating that type five CRS is linked with worse outcomes in septic patients than those without the syndrome [4,5,30]. Based on hospitalization outcomes, the study population was divided into two groups (survivors and deaths) matched by age and gender, enabling an examination of the impact of observed clinical and laboratory parameters. Considering the observed clinical parameters, the group with a negative outcome had low-normal SBP and a higher APACHE II score (equivalent to an average predicted mortality rate of 69.3%) [6]. These findings are consistent with evidence from clinical studies, which shows that lower sBP and higher APACHE II scores are negative predictors of clinical outcomes in septic patients with type five CRS [12,20].

As expected, the study groups differed in terms of duration of hospital stay, as the mortality caused a shorter follow-up period. The average duration of hospital stay in the group with an adverse outcome was 12 days, emphasizing the importance of prompt detection and intensive therapy for these patients [31]. Laboratory findings, on the other hand, showed no significant difference between the groups, suggesting that observed laboratory parameters have a relative value in patients with type five CRS. Because of the syndrome's sudden onset and fast progression, systemic antimicrobial and resuscitative treatment is immediately required. According to the study findings, early antimicrobial and supportive treatment improves the short- and long-term prognosis of such patients [31,33].

The observed laboratory parameters have no statistically significant influence on our patients' hospitalization outcomes, underlining the complexities of type five CRS, where individual clinical criteria and laboratory biomarkers cannot accurately predict the outcome [13]. This could serve as the foundation for future clinical trials aimed at defining individual or combined biomarkers or clinical algorithms that more accurately reflect the severity of type five CRS, allowing for early initiation of appropriate treatment and monitoring its efficacy [28]. In our study, the only laboratory parameter associated with an unfavorable clinical outcome was a decreased platelet count, which is also a component of the DIC scoring system. This condition is very common in critically ill septic patients and is a reliable predictor of poor hospitalization outcomes [20, 42,43].

The parameters that differed across the study groups demonstrated a significant association with adverse clinical outcomes. Higher APACHE II scores reveal the severity of type five CRS and are an easy, accessible, and clinically relevant tool in the mICU settings [6]. Conversely, low-normal or lowered systolic blood pressure and shorter hospital stays were also linked with poor outcomes, suggesting that hypotension and shorter hospital stays, mainly due to fast disease progression, are associated with a higher risk of poor outcomes [44]. Sustained low vascular tone is a signal of the continued presence and activity of the inflammatory mediator storm, and it is the most critical clinical predictor of hospitalization outcomes. Only hemodynamically stable patients experience controlled disease and respond positively to therapy, thereby offering a higher probability for a positive hospitalization outcome [23,25]. The availability and ease of use of these parameters in clinical practice are crucial for timely therapy and monitoring of their effects in septic patients with type five CRS.

Despite clinically confirmed cardiac dysfunction, measured EF did not differ significantly between survivors and non-survivors, implying that diastolic dysfunction, which was not specifically investigated in this study, may play a more significant role than previously assumed [23,24,44-47]. Sepsis-induced myocardial depression often includes impaired ventricular relaxation and elevated filling pressures, which can exacerbate renal congestion and reduce renal perfusion. Several echocardiographic studies have shown that diastolic dysfunction in sepsis is associated with worse outcomes, independent of systolic function, and may be an under-recognized driver of multi-organ injury in CRS‑5 [39, 48]. This suggests that future research should incorporate a systematic assessment of diastolic function in this patient population. Although one-third of patients received specialized hemodialysis therapy (CytoSorb), the clinical outcomes remained unchanged. This finding is consistent with recent meta-analyses and systematic reviews, which indicate that while CytoSorb hemoadsorption can effectively reduce circulating cytokine levels and may transiently improve hemodynamic parameters, it has not demonstrated a consistent mortality benefit in unselected septic populations [40,49]. Possible explanations for the lack of impact include heterogeneity in patient selection, timing of therapy initiation, and severity of illness at the start of treatment. Our results align with these observations, underscoring the need for further studies to define optimal patient selection and timing for CytoSorb use in CRS‑5. This raises concerns regarding the most appropriate timing and patient selection for this therapy [34,49,50,51]. However, preventative actions are even more important to prevent these conditions from occurring and, if they do, to treat them promptly and effectively [3].

In our study, the most common pathogens responsible for septic conditions were *Staphylococcus epidermidis*, coagulase-negative *Staphylococcus*, *Staphylococcus aureus*, and *Acinetobacter* spp. Formulating local antimicrobial guidelines for individual mICUs is crucial for selecting initial antimicrobial agents, controlling drug resistance, and reducing mortality [52]. The study's limitations include the size of the examined population and an inconsistency in the timing of treatment interventions. Furthermore, the heterogeneity in the time-consuming process from the symptom emergence to mICU admission and antimicrobial treatment start may have a considerable influence on clinical presentation and hospitalization outcomes of the observed patients.

The use of extracorporeal membrane oxygenation (ECMO) at our institution, as well as many other hospitals in Serbia, is severely restricted. If the patient's condition necessitates the use of ECMO, he or she must be transported to another facility, facing further transportation risks. That does not preclude the use of ECMO for patients suffering from high-risk CRS-5.

In the current study, the use of ECMO was planned for several patients; however, it was not implemented due to the patients' life-threatening condition and the predicted high-risk transportation. All of them survived with the other recommended medical care. Unfortunately, some other patients did not receive ECMO treatment due to an early adverse outcome.

## Conclusions

In our study, we analyzed the prevalence and clinical outcomes of type five CRS in patients with sepsis in the mICU. The high mortality rate (66%) confirms the severity of this clinical condition. At the same time, key factors associated with poor outcomes were lower SBP, higher APACHE II scores, and shorter hospital stays, highlighting the importance of early diagnosis and intensive therapeutic intervention. Thrombocytopenia was the only parameter in this study that differed between patients with different hospitalization outcomes. Lower platelet counts were observed in patients with poor outcomes, suggesting a potential role in predicting unfavorable prognosis in septic patients.

The research conducted confirms that patients with sepsis and type five CRS have significantly worse prognoses, emphasizing the importance of timely recognition and aggressive therapeutic intervention. A limitation of this observational cohort study is the relatively small number of participants, the absence of a control group, and the impossibilities and inadequacies of APACHE II scores, as well as the extremely restricted availability of non-standard CRS-5 phenotyping at our institution and in Serbia. In line with that, we concentrated on the set of variables accessible in any middle-income country's national health system. This contributes to the model's practical use in underdeveloped health systems. To prevent the influence of too many confounding variables, we carefully defined explicit inclusion and exclusion criteria. However, it provides a solid foundation for future interventional studies focused on exploring combinations of biomarkers or developing predictive models for type five CRS, aimed at reducing morbidity and mortality.
